# Shrimp Waste Upcycling: Unveiling the Potential of Polysaccharides, Proteins, Carotenoids, and Fatty Acids with Emphasis on Extraction Techniques and Bioactive Properties

**DOI:** 10.3390/md22040153

**Published:** 2024-03-28

**Authors:** Nicola Rossi, Clara Grosso, Cristina Delerue-Matos

**Affiliations:** REQUIMTE/LAQV, Instituto Superior de Engenharia do Porto, Instituto Politécnico do Porto, Rua Dr. António Bernardino de Almeida 431, 4249-015 Porto, Portugal; rossinicola93@gmail.com (N.R.); cmm@isep.ipp.pt (C.D.-M.)

**Keywords:** shrimp waste, green extraction, green chemistry, chitin, chitosan, polysaccharides, protein, lipids

## Abstract

Shrimp processing generates substantial waste, which is rich in valuable components such as polysaccharides, proteins, carotenoids, and fatty acids. This review provides a comprehensive overview of the valorization of shrimp waste, mainly shrimp shells, focusing on extraction methods, bioactivities, and potential applications of these bioactive compounds. Various extraction techniques, including chemical extraction, microbial fermentation, enzyme-assisted extraction, microwave-assisted extraction, ultrasound-assisted extraction, and pressurized techniques are discussed, highlighting their efficacy in isolating polysaccharides, proteins, carotenoids, and fatty acids from shrimp waste. Additionally, the bioactivities associated with these compounds, such as antioxidant, antimicrobial, anti-inflammatory, and antitumor properties, among others, are elucidated, underscoring their potential in pharmaceutical, nutraceutical, and cosmeceutical applications. Furthermore, the review explores current and potential utilization avenues for these bioactive compounds, emphasizing the importance of sustainable resource management and circular economy principles in maximizing the value of shrimp waste. Overall, this review paper aims to provide insights into the multifaceted aspects of shrimp waste valorization, offering valuable information for researchers, industries, and policymakers interested in sustainable resource utilization and waste-management strategies.

## 1. Introduction

Global shrimp production reached 5.6 million tons in 2023 [1] and is projected to increase to 7.28 million tons by 2025, exhibiting a compound annual growth rate (CAGR) of 6.1% from 2020 to 2025 [2]. The top five producers of shrimp in 2023 are Ecuador (first position), China (second position), India (third position), Vietnam (fourth position), and Indonesia (fifth position), with Asian countries being major contributors to shrimp farming, accounting for more than 80% of global shrimp production [1,3]. The most popular shrimp species consumed worldwide are *Litopenaeus vannamei* Boone (syn. *Penaens vannamei* Boone, whiteleg shrimp), *Penaeus monodon* Fabricius (giant tiger prawn), and *Macrobrachium rosenbergii* de Man (giant river prawn) [4]. Due to their common presence in both Atlantic and Mediterranean Sea, the species *Cragon crangon* L. (brown shrimp), *Palaemon serratus* Pennant (common prawn), and *Palaemon varians* Leach (common ditch shrimp) also have to be considered [5].

The shrimp processing industry produces around 3.8 million tons of waste worldwide annually, constituting approximately 50 to 60% of the catch volume [6], taking into account that, in the case of shrimps, the head and tail portions may collectively represent 45 to 60% of the total weight. While a limited amount of shrimp waste is employed as animal feed and incorporated into aquaculture feed formulations [6], most of it ends up being disposed of, and disposing of this waste is not only unprofitable but it can also represent an environmental problem if not properly handled [7,8]. Currently, the primary disposal method for shrimp processing waste involves landfilling and ocean dumping, causing alterations to soils, water, and marine ecosystems. Moreover, this approach may lead to the release of unpleasant amine gases during the fermentation of shrimp waste, rendering nearby areas inhospitable. This poses a substantial environmental challenge, contributing to pollution and potentially endangering the ecological environment. Additionally, the washing and cooking processes of shrimp contribute to wastewater pollution, generating approximately 1 L of wastewater per Kg of cooked shrimp [9].

**Figure 1:**
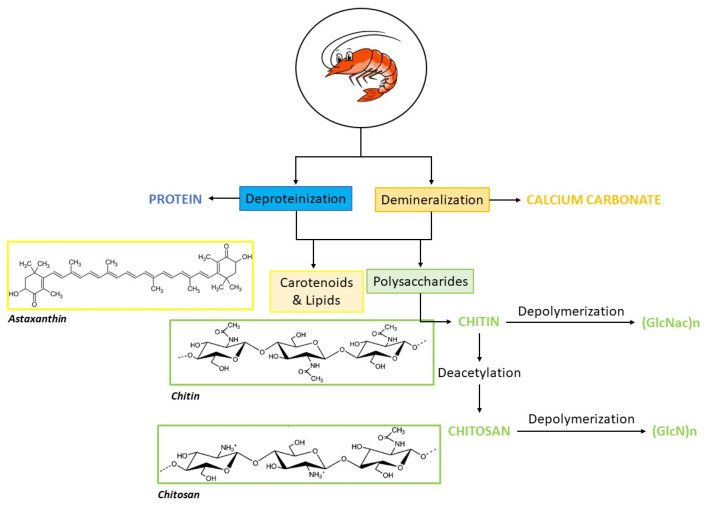
Classes of compounds present in shrimp shell with industrial applications (adapted from [3]).

In addition, the indiscriminate disposal of processing scraps also leads to the waste of valuable resources on a large scale. It is noteworthy that this waste contains several useful bioactive compounds (Figure 1) such as proteins, peptides [10], polysaccharides (chitin and its derivative chitosan) [11], pigments (mostly carotenoids) [7], enzymes [12], lipids [13], minerals (especially calcium, potassium, sodium, and zinc) [14], and vitamins [15].

The amount of each constituent depends on sources and processing conditions [3]. These bioactive compounds have a wide range of applications: as raw material for pet food [16] and as isolated compounds in medicine, in pharmaceutics [3], for cosmetics [17], food and nutrition [18], various sectors in manufacturing (the paper [19] and textile [20] industries, for example), in biotechnology [3], and as food additives [21]. Moreover, these compounds can also function as components integrated into sought-after seafood products.

**Figure 2:**
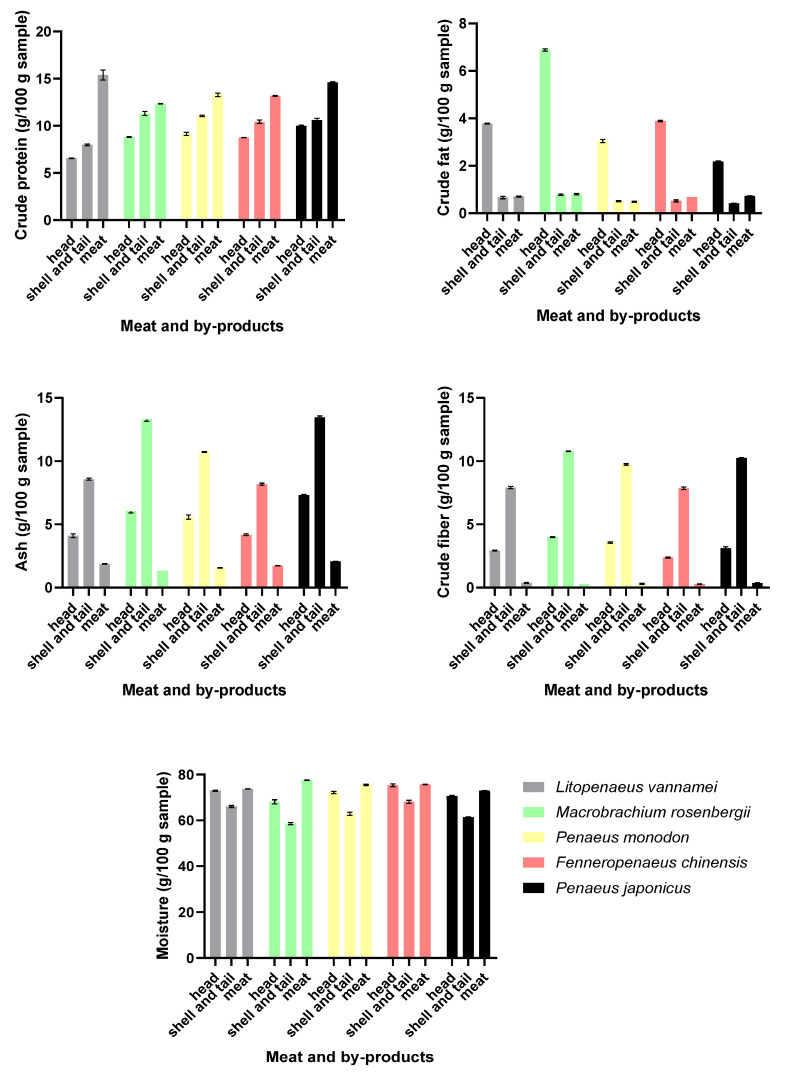
Proximate components of shrimp meat and byproducts (g/100 g sample) [22].

Liu et al. [22] compared the proximate composition of meat and byproducts (heads, tails, and shells) of five shrimp species, namely *Litopenaeus vannamei*, *Macrobrachium rosenbergii*, *Penaeus monodon*, *Fenneropenaeus chinensis*, and *Penaeus japonicus*. The authors found that crude protein was found in the greatest abundance in the meat, while crude fat was mainly found in the heads. Shells and tails contained the highest amount of ash and crude fiber, and, depending on the species, the highest moisture content was found in the heads or the meat (Figure 2).

Besides shrimp wastes, which are rich in valuable compounds, shrimp wastewater, comprising a mixture of organic matter, uneaten feed, excretions, and other waste materials from shrimp farming operations, is produced at a large scale worldwide. This wastewater poses environmental challenges if not managed properly. One of the key concerns associated with shrimp wastewater is its high organic content, which can lead to oxygen depletion in water bodies if released untreated. This oxygen depletion can harm aquatic life and disrupt ecosystems. Additionally, shrimp wastewater may contain excess nutrients such as nitrogen and phosphorus, which can contribute to eutrophication, a process that results in algal blooms and subsequent depletion of oxygen levels. A good number of new technological approaches have been used for wastewater management, including caviation, high-rate algal pond system, use of nanomaterials, biofloc technology, and nanoadsorbent and polymeric nanoadsorbents; this subject was recently reviewed by Iber and Kasan [23].

## 2. Composition of Shrimp Shell

Understanding the composition of shrimp shell is important as a significant portion of the extracted and recovered material originates from it. Shrimp shells comprise densely woven matrices of chitin fibers intertwined with proteins. These matrices gain reinforcement through the deposition of mineral salts, primarily calcium salts [24,25]. Pigments and lipid compounds are also present in the shells, with the quantity and characteristics varying among species and individuals based on factors like growth stage, gender, feeding, and environmental conditions.

**Figure 3:**
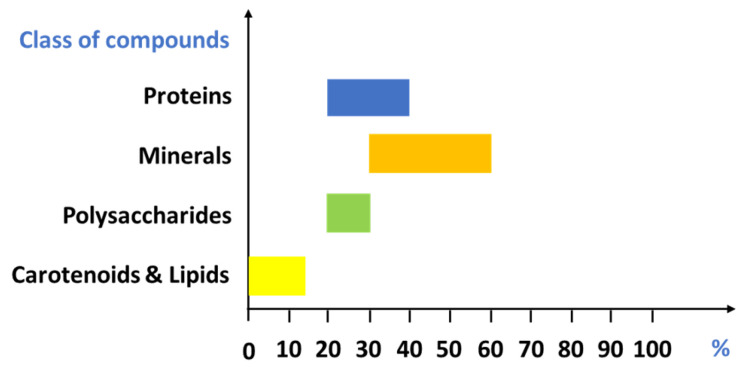
Average starting composition of shrimp shell before industrial processing.

Different biological species exhibit structural variations, resulting in broad ranges of chitin, protein, mineral matter, and pigments percentages in crustacean shells (Figure 3). Typically, exoskeletons consist of approximately 20–40% proteins, 30–60% minerals (predominantly calcium carbonate), 20–30% polysaccharides, and 0–14% other compounds such as pigments (e.g., astaxanthin) and lipids (muscle residues and carotenoids) [26,27].

## 3. Polysaccharides

Polysaccharides are biological molecules composed of long chains of monosaccharides. They exist in countless forms in nature and their function is to provide energy and structural support to cells. The polysaccharides found in shrimp waste are primarily chitin and chitosan, serving a structural role within the crustacean’s exoskeleton. Chitin and chitosan have important commercial applications and are mostly derived from the exoskeletons of crabs, shrimp, shellfish, and lobsters; these materials represent significant byproducts of the seafood industry [28].

The economic value (Table 1) of these polymers is strictly linked to their properties, such as ready availability, compatibility with biological systems, biodegradability, immunological, non-toxicity, antimicrobial properties, the ability to chelate heavy-metal ions, gel-forming capabilities, ease of chemical modification, and a strong affinity for proteins, among others [29].

The next sections will focus on two polysaccharides, chitin and chitosan, concerning their properties and applications.

### 3.1. Chitin

Chitin, from the Greek words χιτών (khitōn), which means tunic or envelope, is a polysaccharide that represents 20 to 30% of shrimp shell [53].

Discovered in 1821 by the French pharmacist Henri Braconnot, chitin is the second most abundant biopolymer on earth, after cellulose [54], with which it has a structural similarity: chitin has an acetamide group (CH_3_-CO-NH_2_) at C-2 in place of the hydroxyl group (-OH) in cellulose [55]. Besides its presence in the exoskeleton of insects and other arthropods, chitin can also be found in the cell wall of fungi and yeasts, in the perisarc of hydrozoans, and in the epidermal cuticle and other superficial structures of many other invertebrates [56,57].

As shown in Figure 1, the chemical structure of the chitin polymer consists of a linear chain of 1→4 linked 2-acetoamido-2-deoxy-β-D-glucopyranose units [58]. Chitin is classified as α, β, and γ-chitin according to anti-parallel, parallel, and alternated alignments of the polymeric chains, respectively. α-Chitin is usually extracted from crustacean exoskeletons, mainly from shrimps and crabs; β-chitin can be isolated from squid pens; and γ-chitin is obtained from fungi and yeasts. Crustacean shells and fungal mycelia are industrial sources of chitin [59].

### 3.2. Chitosan

Chitosan is a linear polysaccharide and is commercially produced by the deacetylation of chitin. It is also the only known natural cationic polysaccharide [60].

Chitosan possesses one primary amino and two free hydroxyl groups per C6 building unit [61]. Due to the ready availability of free amino groups, chitosan carries a positive charge, enabling it to interact with negatively charged surfaces/polymers and undergo chelation with metal ions such as Ni^2+^, Zn^2+^, Co^2+^, Fe^2+^, Mg^2+^, and Cu^2+^, especially under acidic conditions. This high chelating capacity has led to its widespread application in the removal or recovery of metal ions in diverse industries [62].

While chitosan is a weak base and insoluble in water and organic solvents, it exhibits solubility in dilute aqueous acidic solutions (pH < 6.5). This acidity converts the glucosamine units into a soluble form, R–NH_3_^+^ [63,64]. Chitosan precipitates in alkaline solutions or with polyanions and forms a gel at lower pH. It also serves as a flocculant for wastewater treatment [65].

Commercial chitosan exists in the form of dry flakes, solutions, and fine powder. It has an average molecular weight ranging between 3800 and 20,000 Da and is deacetylated from 66 to 95% [66]. Key characteristics such as particle size, density, viscosity, degree of deacetylation, and molecular weight play crucial roles in influencing the properties of pharmaceutical formulations based on chitosan [67,68,69].

Chitosan’s desirable properties, including biodegradability, low toxicity, and excellent biocompatibility, render it well-suited for applications in biomedical and pharmaceutical formulations [67], support material for gene delivery [31], cell culture [37], and tissue engineering [35,36]. Particularly in these sectors, chitosan and its derivatives find extensive use, thanks to their diverse biological functions, including antimicrobial [70,71,72,73,74,75,76,77], antioxidant [72,73,75], immunostimulant [78], antitumor [77], and antidiabetic [79] effects, among others.

Various techniques for extracting chitin and chitosan from different crustaceans have been documented. Two main extraction procedures are reported, namely chemical and microbial extractions.

### 3.3. Extraction Processes

#### 3.3.1. Chemical Extraction

Chemical extraction techniques have conventionally been employed to extract chitin and chitosan from sources such as crustacean shell waste, insects, fungi, and mollusks. The typical chitin extraction process from marine waste involves demineralization (removal of inorganic CaCO_3_) and deproteinization using strong acids and bases, respectively. A third step of deacetylation in alkali conditions is needed to obtain chitosan [80,81,82,83,84].

Concerning the demineralization step, acidic conditions (e.g., HCl or CH_3_COOH) are usually employed at temperatures up to 75 °C during several minutes–hours. The mineral content of the shells, temperature, extraction time, particle size, acid concentration, and solute/solvent ratio are the main factors affecting the demineralization treatments. Higher temperature and finer particle sizes accelerate solvent penetration into the chitin matrix. For the deproteinization steps, high temperatures (up to 110 °C) for extended duration (hours to days) have also been used for the removal of proteins with NaOH or KOH. Deproteinization depends on alkali concentration, temperature, and the shell mass/alkali ratio (*w*/*v*). Temperature and the solid/solvent ratio are the most critical factors in this step. Finally, deacetylation with concentrated NaOH or KOH occurs at temperatures up to 130 °C for minutes–hours. The degree of deacetylation, solubility, and the MW of the obtained chitosan is mainly influenced by the molar ratio of chitin to NaOH and temperature [59,72]. An intermediate step of bleaching/decoloration to remove pigments (e.g., carotenoids) can also be implemented [59].

The alkali and acids used in the extraction process are released as chemical wastage, contributing to environmental damage due to the significant amounts of chloride, calcium, and sodium [85]. Similarly, side products such as minerals and proteins obtained from shell wastes during chemical extraction often cannot be repurposed for animal feed or fertilizer [26]. Moreover, the deproteinization process yields numerous proteins, peptides, and chitooligosaccharides, which are not utilized for further processing due to the presence of alkali solvents [72].

#### 3.3.2. Green Extraction of Polysaccharides

Despite the previously mentioned chemical method being the most widely used for extracting chitin and chitosan from shrimp waste, various alternative methods with reduced environmental impact are discussed in the literature, regarding green extraction of chitin and chitosan from shrimp waste. Foremost among these is the microbiological method [86,87,88], which has been extensively demonstrated to be more efficient in terms of yield compared to the chemical approach. There are, however, other methods: the microwave-assisted method [89], the ultrasound-assisted method [90,91], the ionic liquid method [92], the subcritical water method [93], the electrochemical method [94], the pulsed-electric-field method [95], the natural deep eutectic solvent method [96], and the enzyme-assisted method [97]. Among these, only a handful of methods are economically viable, environmentally friendly, and pose minimal toxicity to the environment.

##### Biological Extraction (Microbial Fermentation)

As previously discussed, the chemical extraction process can impact the physicochemical properties of chitin and chitosan [98]. Alternatively, efforts have been focused on minimizing chemical treatments by adopting more environmentally friendly processes. Lactic-acid-producing bacteria (e.g., with *Lactobacillus plantarum* subsp. *plantarum* ATCC 14917 and *Bacillus subtilis* subsp. *subtilis* ATCC 6051 [86]) are commonly employed for the demineralization step, while proteases-producing bacteria (e.g., *Streptomyces* sp. SCUT-3 [87], *Paenibacillus mucilaginosus* TKU032 [99], *Paenibacillus elgii* TKU051 [100], *Brevibacillus parabrevis* TKU046 [101]) are used to remove proteins. The deproteination step is promoted by the proteolytic enzymes secreted during microbial fermentation by protease-producing bacteria and, during this step, other valuable compounds are also obtained, namely proteases [99,100,101,102], prebiotics [101,102], and antioxidants [103]. For instance, microbial extraction with *Lactobacillus plantarum* demonstrated a 40% improvement of chitin performance over chemical extraction methods [104]. The development of a three-step microbial fermentation process using *Serratia marcescens* B742, *Lactobacillus plantarum* ATCC 8014, and *Rhizopus japonicus* M193 resulted in the extraction of low-MW chitosan (512.06 kDa) compared with the chemical extraction approach (1959.45 kDa) [105]. Tan et al. [106] employed *Lactobacillus acidophilus* in a single-step fermentation technique to extract chitin from shrimp waste. Protease-producing bacterial strains, including *Pseudomonas aeruginosa*, *Serratia marcescens*, and *Bacillus pumilus*, were employed to extract chitin from shrimp waste. *P. aeruginosa* demonstrated the highest rates of deproteinization (74.76%) and demineralization (78.46%), while the lowest rate was observed for the treatment with *S. marcescens* [107]. Additionally, protease from *Alcaligenes faecalis* and lactic-acid-producing bacterial strains (*Bacillus coagulans*) were used for chitin extraction from *L. vannamei* shell wastes [108]. Another study utilized the protease-producing strain *P. aeruginosa* for extracting chitin from shrimp shell waste [109]. Overall, the proteolytic enzymes produced by bacterial communities have demonstrated superior yields, making this biological extraction process a promising alternative to alkali extraction.

As explained above, the chemically processed final product typically contains a substantial amount of proteins and minerals as impurities [110]. Consequently, the obtained byproducts are not suitable for various applications, such as feed or fertilizer, due to the presence of corrosive chemicals. In contrast, deproteinization achieved through microbial fermentation produces valuable ingredients that can be utilized in animal nutrition while being environmentally safe. Although the bacterial fermentation process may require more time than other methods, it eliminates the need for toxic chemicals.

The fermentation of chitin can be accomplished using carbon sources that are available in agro-food waste (vegetable or fruit waste) [86]. This approach reduces the reliance on corrosive chemicals in the reaction process by employing microbial species in the fermentation technique. Additionally, it helps prevent the biomagnification of toxic chemicals from entering the environment and other living organisms. However, the biological extraction process is currently limited to small-scale production. The major challenges in scaling up from laboratory scale to industrial scale include the need for well-equipped bioreactors for microorganism growth, strain potency, controlled temperature, inoculum concentration, pH, and substrate. The fluctuation in pH can affect the growth of organisms used in fermentation and aging time can influence chitin depolymerization. Fermentation parameters vary depending on the source and quantity of aqua-industrial waste used for fermentation. Moreover, upscaling requires additional energy sources, controlled bioreactors, and highly skilled personnel [111].

Table 2 summarizes the advantages and disadvantages of the chemical and biological extraction approaches.

##### Enzyme-Assisted Extraction (EAE)

In the current context, the enzymatic-assisted extraction method is gaining popularity due to its environmentally friendly nature, making it one of the preferred green extraction methods. Considering growing safety and environmental concerns, the functionalization of natural polymers using enzymes is continuously explored as an attractive and alternative approach to toxic, environmentally hazardous, and nonspecific chemical methods. EAE is more specific, acts rapidly, and reduces the use of energy, chemicals, and/or water compared to conventional processes [112]. It enables the recovery of high-value-added products, including chitin, pigments, or peptides.

Hongkulsup et al. [97] tested a protease-assisted extraction of chitin from shrimp shells after demineralization by organic acid. They achieved a maximum demineralization of 98.64% for lactic acid and 97.57% for acetic acid. The protease (from *Streptomyces griseus*) exhibited a total protein removal efficiency of 91.10%, leading to chitin with lower crystallinity and a higher degree of acetylation compared to chemical extraction.

Marzieh et al. [113] tested a combination of lactic acid (for the demineralization step), trypsin, and ficin (for enzymatic deproteinization), followed by mild-alkali treatment. They obtained 92% deproteinization efficiency. Poonsin et al. [114] used albacore tuna spleen trypsin to extract carotenoproteins from Pacific white shrimp shells. Albacore tuna trypsin showed a similar recovery efficacy of protein or carotenoids, compared with trypsin from bovine pancreas.

Other enzymes, such as papain [115], pepsin [116], and alcalase [117,118], have also been employed for the deproteinization step.

##### Microwave-Assisted Extraction (MAE)

Microwave technology is extensively employed in the food and chemical industries for diverse applications. Various reports highlight its potential to accelerate chemical reactions, enhance reaction yield, and improve product purity and properties compared to conventional heating methods [119,120]. This technology offers advantages such as short heating times, energy efficiency, low process costs, easy process control, and selective heating capabilities [121]. The mechanisms involved in microwave heating include dipolar polarization and ionic conduction [122], leading to thermal effects through inverted heat transfer, a homogeneous microwave field within the sample, and the selective absorption of radiation by polar substances [123]. Microwave irradiation facilitates the direct and rapid transfer of energy into biomass substrates and catalysts, thereby enhancing reaction efficiency [124].

Microwave heating has been employed in the extraction of polysaccharides, including chitosan and cellulose, resulting in reduced chemical usage [125,126,127]. Optimizing process conditions is crucial to enhance the reaction rate and reduce production costs [128]. Various operating conditions, such as reaction time, solvent concentration, and solid-to-liquid ratio, impact the degree of deacetylation and molecular mass of the obtained chitosan [129].

Recent studies have explored the efficiency of microwave technology for chitin and chitosan extraction. For instance, MAE achieves 82% deacetylation of chitosan in 24 min, significantly outperforming conventional heating, which requires 6–7 h for similar deacetylation levels. Both methods yield products with similar structures, morphologies, and chemical compositions. However, chitosan from microwave heating exhibits a higher molecular weight and nearly the same crystallinity as commercial chitosan in a shorter heating time than the conventional method [130]. Moreover, the production of glucosamine from the hydrolysis of chitosan (microwaves combined with alkali conditions) required significantly reduced heating temperature and time compared to conventional hydrolysis. FT-IR results confirmed that the absorption bands of the prepared glucosamine hydrochloride meet the standard compound [131].

Zhang et al. [132] applied the traditional chemical processes for the demineralization, deproteination, and deacetylation steps of shrimp shells; then, they investigated the impact of microwave heating time, H_2_O_2_ concentration, and the solid-to-liquid ratio on the degree of deacetylation and molecular weight of chitosan. The goal was to obtain a low-molecular-mass chitosan with high degree of deacetylation because it has excellent bioactivity including antioxidant, antibacterial, and encapsulation properties. According to the Box–Behnken model, microwave conditions of 80 s of heating time, coupled with a concentration of 1.5% H_2_O_2_ and a 1:40 solid-to-liquid ratio, yielded the highest degree of deacetylation (90.58%) and the lowest molecular weight (124.25 kDa) of chitosan. Chitosan obtained through microwave techniques exhibited higher antibacterial activity than chitosan with high molecular weight and a degree of deacetylation of 52.14% (obtained by conventional chemical extraction).

High power in microwave-assisted extraction systems requires low alkali concentration for the extraction process. El Knidri et al. [89] investigated the effect of reaction time with HCl and NaOH concentrations at different powers (90 W, 160 W, 350 W, 500 W, and 650 W) using microwave heating for chitosan extraction. Using a 50% NaOH solution in a power range of 500–650 W resulted in a low degree of acetylation, with deacetylation exceeding 80% after 10 min. MAE allowed the production of chitosan with medium and high molecular weights (300–360 kDa).

The aforementioned studies collectively underscore the significant reduction in heating temperature and time achieved through microwave technology in the entire chitosan extraction process, surpassing conventional methods. The deproteinization process under microwave heating yields a lower residual protein (11.4%) in a shorter duration, whereas the conventional heating method for 2 h results in 11.7% residual protein. Optimizing the reaction setup holds the potential to enhance chitosan yield and efficiency [133]. Consequently, the implementation of MAE technology on an industrial scale could lead to substantial energy savings and prove to be an economically viable extraction method [133]. Although, one of the drawbacks of MAE is the scale-up of the process due to the complex mass transfer involved, as addressed by different authors [134,135]; there are already some industrial MAE systems produced by SAIREM company (https://www.sairem.com/industrial-scale-continuous-microwave-assisted-plants-extraction/, accessed on 21 March 2024).

##### Subcritical Water Extraction (SWE)

Subcritical water treatment holds immense potential for generating raw materials from waste biomass, concurrently reducing waste stream volumes. This method offers several advantages, including rapid reaction rates and the substitution of acids/bases with a more environmentally acceptable solvent. Subcritical water hydrolysis typically occurs at temperatures between 100 °C and below water’s critical temperature (Tc = 374 °C) under sufficient pressure (below Pc = 22.1 MPa) to maintain water in a liquid state [133].

Due to its environmentally friendly characteristics and higher abundance, water is predominantly utilized in subcritical reactions for processing agricultural biomass residues. Pressurized subcritical water, ranging between 100 and 374 °C, serves as an exceptional solvent for various cellulosic and proteinaceous biomasses. At elevated subcritical water temperatures, chitin undergoes hydrolysis, oligosaccharides decompose, and compounds such as amino acids and fatty acids are recovered. This method also enables the modification of chitin morphology for enhanced enzymatic digestion or protein removal from crab shells, along with the extraction of mineral elements, including calcium and citric acid [133].

Recent investigations have employed subcritical treatment for chitin and chitosan extraction, as discussed below. Espíndola-Cortés et al. [93] employed subcritical water treatment to extract calcareous chitin from shrimp, achieving 96% deproteinization. The optimization of experimental conditions using the Taguchi design increased the crystalline domain size in chitin fibers. The highest α-chitin content (82.2 wt%) was obtained with a 0.17 chitin/distilled H_2_O (wt/wt) ratio after a 30 min treatment at 260 °C and 5 MPa. Chemical, structural, and morphological characterizations demonstrated complete protein removal from the chitin matrix under subcritical conditions within a short reaction time.

##### Ultrasound-Assisted Extraction (UAE)

Ultrasound’s cavitation effect plays a crucial role in increasing the solubility of proteins associated with chitin. This effect may be attributed to the depolymerization of macromolecules, the dissociation of covalent bonds in polymer chains, and the dispersion of aggregates [136]. High-intensity ultrasound signals, operating at 750 W power and 20 kHz ± 50 Hz frequency, enhance the efficiency of chitin extraction, reducing the extraction time and eliminating the need for high temperatures [90].

Dipole–dipole interactions on biopolymer chains significantly influence the polymer’s crystallinity, creating a hindrance to chitinase-mediated hydrolysis for obtaining chitin. To overcome this, sonication and steam explosion (SE) are considered effective pretreatments. This approach, applied to chitin oligosaccharide by partially purified chitinases from *Lecanicillium lecanii* [137], results in decreased crystallinity indices and average degree of acetylation in chitin samples. SE treatment, despite causing slight depolymerization through sonication, profoundly influences amino-based hydrogen bonding in the samples. It reduces hydrogen bonding after SE treatment without reducing sugars at the initial stages of reactions, making SE less chemically destructive than sonication. Consequently, depolymerization-free enhanced enzymatic hydrolysis with minimal acetylation can be achieved through SE pretreatment.

Additionally, ultrasound irradiation and thermochemical extraction were compared for the conversion of α-chitin to chitosan. The reaction medium underwent high-intensity ultrasound irradiation in association with nonisothermal conditions [138]. The study examined the effects of heat application in the reaction medium (100 °C–105 °C) and high-intensity ultrasound (45 min and 60 min) on the recovery of chitosan. Results indicated that the α-chitin suspension subjected to nonisothermal ultrasound-assisted N-deacetylation for a longer period yields more chitosan (99.4% for 60 min and 95.3% for 45 min) with a lower degree of acetylation (29.0% for 60 min and 31.0% for 45 min) [138].

Introducing ultrasound irradiation at regular intervals (7.5 min/period with non-sonication for 5 min/period) enhances chitosan yield by 100%, accompanied by the lowest degree of acetylation of 22.1% [138].

The conversion of chitin into chitosan through unidirectional ultrasound of a single frequency at low temperatures and short reaction times (<30 min) proves to be inadequate for practical applications due to its inhomogeneous mechanical (sound) field. Ultrasound leads to higher chitin degradation, with these degraded molecules being washed out during the washing process. Improved demineralization and deproteinization can be achieved through chemical treatment assisted by ultrasonication [139]. Likewise, a prolonged reaction time (ultrasound for 60 min) on the α-chitin suspension, with a resting period of 25 min, increased chitosan content by 99.9%. However, it has been identified that high-intensity UAE leads to severe depolymerization [138], necessitating further exploration.

##### Chitosan Purification

Concerning the purification of chitosan isolated from shrimp [140] and other crustaceans’ shells [141], a three-step process is usually employed. The first step consists of the removal of insolubles through filtration, followed by a second step of reprecipitation of chitosan with 1N NaOH. After, a demetallization of retrieved chitosan is performed by using solutions of sodium dodecyl sulfate and EDTA. Sodium dodecyl sulfate can remove any protein residuals that can be present in the crude chitosan product, while EDTA is used as a metal chelating and demetallizing agent as well as a mild acid in the purification process [141]. Other purification techniques also include adding an alkali metal salicylate such as sodium salicylate followed by placing the mixture in an ice bath; this precipitates the chitosan which can then be separated by centrifugation, or only physical separation using ultrafiltration and molecular sieves [141]. Chemical processes can be time-consuming and increase the potential chemical contamination in the final product as more chemicals are introduced in the process [141]. Therefore, whenever possible, physical methods should be adopted.

##### Energy Consumption and Emission in Green Extraction

The conventional pulping method for chitin production from marine shell waste in many developing countries generates significant waste effluent, raising environmental health concerns. This process results in substantial CO_2_ emissions and requires substantial energy and coal fuel consumption. Green extraction techniques have been introduced to address these issues. For instance, betaine hydrochloride (BCl), an organic and biodegradable solvent, has shown promise in chitin hydrolysis, offering a potentially better alternative to HCl. Physical methods like instant catapult steam explosion (ICSE) and electrochemical techniques are also explored to reduce CO_2_ emission and energy consumption during extraction. Integrating such methods could provide a sustainable solution to energy challenges [142].

However, green technologies also present some drawbacks concerning energy costs. For instance, for pressurized extraction processes, a lot of energy is required to convert the solvent to the subcritical or supercritical state, making SWE or CO_2_-supercritical fluid extraction techniques highly costly [143].

##### Future Directions

Recent advancements in extraction techniques have shown a gradual improvement in sustainability, particularly in energy consumption and greenhouse gas emissions. The use of biobased chemicals in conjunction with physical processes has demonstrated positive effects. Water, as a green solvent, has gained prominence due to its non-toxic nature and increased solute hydration properties. Additionally, the thermal properties of chitin play a crucial role in various material applications, necessitating careful consideration during the purification stage. The upscaling of lab-scale methods to industrial levels remains a challenge, particularly for techniques such as high-pressure CO_2_ extraction and MAE. Addressing these challenges and further research on the impact of chemicals in the green extraction process, especially in medicinal chemistry applications, are essential for the development of sustainable and environmentally friendly practices.

### 3.4. Applications of Polysaccharides

Considering the wide range of applications of polysaccharides, describing them all in one article would be impractical. In this section, both the main uses and potential future applications of particular interest will be analyzed; a highlight will be placed on bio-pharmaceutical applications. This bio-pharmaceutical interest is due to both the aforementioned intrinsic properties of these polysaccharides (biocompatible, biodegradable, and non-toxic) and the fact that glucosamine, the primary constituent of chitosan, is a naturally occurring substance synthesized within the body from glucose. It plays a crucial role in the production of glycosaminoglycan, a key component in the formation of cartilage tissue. Additionally, glucosamine is found in tendons and ligaments. All these factors make this polysaccharide outstanding for bio-pharmaceutical applications.

#### 3.4.1. Anticancer Activity

Chitosan and its derivatives have been reported to selectively permeate through the cancer cell membranes and function as anticancer agents [144]. Table 3 summarizes the mechanisms behind the anticancer activity of chitosan and derivatives.

#### 3.4.2. Potential Drug Carrier: Microsphere

Chitosan has proven to be effective in enhancing the dissolution rate of poorly soluble drugs, offering potential for improving the bioavailability of such medications. Through the reaction of chitosan with controlled amounts of multivalent anions, crosslinking occurs between chitosan molecules. This crosslinking is widely utilized in the preparation of chitosan microspheres. Additionally, other crosslinking agents like glutaraldehyde, formaldehyde, and the naturally occurring genipin have been employed for microsphere preparation. Various methods, including coacervation, multiple emulsion, and solvent evaporation, have been utilized for the preparation of chitosan microspheres, allowing for the modification of particle sizes suitable for oral, nasal, and parenteral drug delivery [153,154].

Upon oral administration, drug-loaded chitosan microspheres dissolve in the gastric medium, liberating the drug initially. Coating chitosan with hydrophobic polymers such as ethylcellulose can stabilize the microspheres. The entrapment efficiency of drugs in chitosan microspheres is influenced by the concentration of chitosan, with higher concentrations leading to increased entrapment efficiency. Drugs can also be loaded using a passive absorption method by adding microspheres to a drug solution, utilizing the swelling properties of the microspheres [155].

The release of drugs from chitosan microspheres is dependent on factors such as the molecular weight of chitosan, chitosan concentration, drug content, and crosslinking density. Chitosan microspheres have been employed to incorporate various therapeutic agents, including anticancer [152,156], anti-inflammatory [157], antibiotic [158,159], antithrombotic [160], and antidiabetic [161] agents, among others, to achieve controlled release.

#### 3.4.3. Blood-Compatible Membrane

Blood compatibility is a crucial property for biomaterials in contact with blood. Upon contact, blood proteins adhere to the surface, triggering biological responses like thrombin formation, platelet activation, and coagulation. To avoid adverse effects, particularly thrombosis, biomaterials should be blood compatible. Surface characteristics such as chemical composition, topography, and charge influence blood compatibility. Materials with nano-fibrous membranes mimicking the extracellular matrix exhibit good blood compatibility. Heparin, a natural anticoagulant, is often mimicked in blood-contacting materials to prevent coagulation. Polysaccharides like chitosan and cellulose derivatives have been modified to mimic heparin. Electrospinning is used to fabricate nano-fibrous materials resembling blood vessel walls. Chitosan, despite its challenges in electrospinning, can be combined with other polymers to overcome this. Nano-fibrous membranes produced through electrospinning have shown promising blood compatibility [162].

Despite these advancements, a significant challenge remains: the issue of surface-induced thrombosis associated with artificial membranes. Current solutions involve heparinization of blood to prevent clotting, but this poses a risk for individuals prone to internal hemorrhage during dialysis.

Therefore, the most pressing challenge in this field is the development of inherently blood-compatible membranes.

#### 3.4.4. Hemostatic Dressing

The establishment of an efficient pre-hospital trauma care system necessitates a wound management approach with rapid hemostatic capabilities, infection inhibition, and enhanced healing support to improve the chances of survival for injured patients. Several hemostatic agents based on chitosan, such as Combat Gauze, Celox Rapid, ChitoGauze, and ChitoFlex, all FDA-approved and commercially accessible, have been developed to mitigate excessive hemorrhaging [163].

Ongoing advancements in the manufacturing processes of chitosan-based dressings aim to augment their hemostatic and antibacterial properties. Integration of antioxidants (e.g., curcumin) or polysaccharides (e.g., cellulose/gelatin) and chemical coupling with chitosan further enhance the mechanical, gas permeability, drug delivery, and wound-healing characteristics of chitosan bandages. Studies exploring applications like gelatin-microsphere-containing tetracycline hydrochloride broaden the scope for research in chronic wound management, especially in diabetic foot cases, by incorporating pharmacologically active ingredients [164].

Chitosan-based composites, known for their pH responsiveness due to amine groups, are gaining popularity in biomedicine. It is evident that, by optimizing the degree of deacetylation, molecular weight, and leveraging grafting technology, one can develop an effective hemostatic agent with the required antibacterial properties. The discussed chitosan nanocomposites exhibit superior properties compared to their counterparts, and the nanoparticles used can be further functionalized to create an environmentally responsive drug-delivery system [164].

#### 3.4.5. Bioimaging

This technique has found extensive applications in both research and clinical trials [165,166,167], enabling the observation and investigation of biological phenomena ranging from subcellular to mammal grade phenomena. Bioimaging, recognized for its potential in elucidating biological problems, possesses the capability to automatically quantify, detect, and profile phenotypic changes [168,169].

Wang et al. [170] developed a dual-enzyme-loaded nanogel for pathological responsive ultrasound imaging and T2-weighted magnetic resonance imaging. This nanogel probe, composed of dual enzyme species, superparamagnetic particles of iron oxide, and glycol chitosan gel with combinatorial properties, enhanced enzymatic reactions and provided a sufficient amount of oxygen bubbles for ultrasound imaging. In vivo results from tumors revealed a 7-fold higher concentration after 1 h of injection. Experimental analyses involved the regular dosing of mice with fabricated nanogels for imaging to demonstrate their in vivo distribution. In another study, chitosan/fullerene nanogels were intravenously injected into nude mice bearing KB tumors [171]. It was reported that, despite the absence of isotopes or fluorophores, chitosan/fullerene nanogels could be adopted for photo-luminescent tumor imaging. These nanogels exhibited highly resoluting fluorescent intensity after 8 h, indicating their potential for in vivo photo-luminescent imaging in various types of malignant tumor cells.

#### 3.4.6. Antioxidant Activity

Reactive oxygen species (ROS), including H_2_O_2_, hydroxyl, and superoxide radicals, contribute to oxidative stress, which is linked to various pathologies such as cancer, cardiovascular disease, respiratory disease, premature aging, rheumatoid arthritis, kidney disease, and inflammation [172]. Chitin, along with compounds like vitamin C and E, has demonstrated antioxidant effects, making it a potential ingredient for functional foods that could help prevent age-related and diet-related diseases [173,174].

The oxidation of highly unsaturated food lipids can result in off-flavor and rancidity. While synthetic antioxidants like butylated hydroxytoluene (BHT) and butylated hydroxyanisole (BHA) are commonly used, concerns about their health hazards have led to a preference for safer and natural alternatives [98].

Hafsa et al. [175] evaluated the antioxidant properties of chitin and chitosan extracted from *Parapenaeus longirostris* Lucas shrimp shell waste. The scavenging ability of chitin, classical deacetylated chitosan, and ultrasound-assisted deacetylated chitosan on DPPH radicals ranged from 4.71% to 21.25%, 11.45% to 32.78%, and 18.27% to 44.17%, respectively, at concentrations of 250 to 1000 µg/mL. The inhibition of lipid peroxidation with thiobarbituric-acid-reacting substances ranged from 11.7% to 51.63%, 17.24% to 63.52%, and 29.31% to 77.39%, respectively, in an interval of 250 to 1000 µg/mL.

#### 3.4.7. Antimicrobial Treatment of Textiles

Textiles serve as a habitat for attached microorganisms, posing a potential threat to human health through disease transmission. Mitigating this risk is crucial, emphasizing the significance of antibacterial treatment in textiles. Chitosan, known for its broad-spectrum antibacterial activity against pathogens like *Staphylococcus aureus*, *Escherichia coli*, and *Bacillus subtilis* [175,176], is a key player in achieving this objective.

Two primary approaches are employed for manufacturing antibacterial textiles using chitosan. The first involves finishing the fabric by affixing chitosan. Moattari et al. [177] employed citric acid as a cross-linking agent and sodium dihydrogen phosphate as a catalyst to graft chitosan onto cotton textiles under UV irradiation, resulting in effective antibacterial properties. The second approach entailed creating antibacterial fibers, which are then woven into fabrics. Japan Fuji Textile Co., Ltd. developed a stable ultrafine chitosan powder with a particle size of approximately 5 μm. This powder was subsequently blended into a viscose solution, leading to the production of fibers named “chitopoly” with remarkable antibacterial properties [178].

#### 3.4.8. Antimicrobial and Stimulator Agents for Agriculture

The bactericidal, fungicidal, and other inherent properties of chitin and its derivatives position them as excellent candidates for agricultural applications. Moreover, they serve as indicators of mold contamination in agricultural products [179]. Chitin-treated seeds, such as wheat, cabbage, pumpkin, chili, and cucumber, have been reported to accelerate growth by introducing chitinous elements into the soil–plant mixture, leading to a noticeable reduction in insect penetration and the presence of pathogenic fungi [180].

Rkhaila et al. [181] showed that chitosan and chitin–chitosan mixture greatly promoted *Lycopersicon esculentum* L., *Capsicum annuum* L., and *Solanum melongena* L. seed germination percentage by 16%, 34%, and 22%, compared to the control, and resulted in an improvement of their vigor index and shoot and root lengths. Weekly soil amendment with chitin or chitosan induced the stimulation of plants parameters (lengths and fresh and dry weights of aerial and root parts). Moreover, a very significant increase in the number and weight of fruits was marked by the weekly soil amendment with the chitin–chitosan mixture at 25 and 100 mg/L.

#### 3.4.9. Biomaterial for Industrial Packaging

Consistent efforts are underway to advocate for the utilization of bio-composite materials across various applications due to their cost-effectiveness, biodegradability, reliance on non-petroleum sources, and minimal carbon emissions. Employing chitosan as a substitute for synthetic polymers in food packaging is particularly valuable due to its antimicrobial and biodegradable properties [182]. Chitosan films, created through wet or infrared drying, show varied properties, with modifications like amide formation enhancing strength and reducing solubility. Chemical modifications, including surface hydrophobicity changes, influence protein adsorption behavior, showcasing the potential for tailored responses to bio-macromolecules. Additionally, blending chitosan with dialdehyde starch enhances mechanical and antimicrobial properties. Chitosan films find applications in food preservation, extending the shelf life of fruits through coatings that inhibit fungal growth. In agriculture, chitosan acts as an active packaging material, gradually releasing preservatives for prolonged storage and, therefore, offer a viable eco alternative to synthetic packaging materials [183].

Elhussieny et al. [184] demonstrated the preparation of chitosan composite films as polymeric matrices, revealing a significant improvement in the thermal degradation temperature of chitosan when rice straw was added. Notably, these composite films undergo degradation, leaving behind zero waste. Bio-based packaging films have been innovatively developed, incorporating liquefied shrimp shell chitin [185]. The application of these chitin-containing biofilms in wrapping cherries and tomatoes extends their shelf life by 10 days. The introduction of beta-cyclodextrin not only enhances the antimicrobial potential of the biofilms but also delays the release of cinnamaldehyde [186].

Al-Ali et al. [187] delved into the properties of chitosan composite film extracted from shrimp, combined with ginger essential oil. Their findings indicate a significant decrease in tensile strength with an increased concentration of ginger essential oil. However, the elongation also exhibits improvement. Studies by Tamer et al. [188] and Kumar et al. [189] suggest that chitosan-based packaging films hold promise for applications in pharmaceutical, cosmetic, and food industries, given their anti-free radical nature. Dasumiati et al. [190] have undertaken the development of bioplastic using *Manihot utilissima* Pohl (cassava) peel and shrimp shells.

## 4. Proteins

Proteins, significant bioactive molecules composed of amino acid chains, stand among the six major classes of nutrients essential for human health and play crucial roles in various biological processes within living organisms. Proteins are involved in the structure, function, and regulation of cells and tissues. Proteins are present in relatively high quantity in the shrimp waste, representing 30 to 40% of the total weight of the waste [191]. The essential amino acids comprises a higher proportion than the non-essential amino acids [87]. The protein present in shrimp waste maintains a close association with chitin and minerals, and proteins derived from the protein–chitin–minerals complex find various applications. Protein hydrolysates obtained from shrimp waste can be utilized in a range of industries such as pharmaceuticals, human nutrition, animal nutrition, and cosmetics [9,18,192,193,194,195]. Additionally, protein hydrolysates serve as a valuable nitrogen source in growth media for microorganisms [196].

### 4.1. Extraction Methods

Like chitin, the extraction of protein from shrimp waste involves a deproteinization process. A brief summary of recent studies on enzymatic-assisted extraction from shrimp wastes is shown in Table 4.

For instance, digestive alkaline proteinases from golden grey mullet (*Liza aurata*) have optimal activity at pH 8.0 and 60 °C. The crude proteases proved effective in the deproteinization of shrimp wastes, with protein recovery reaching about 76% after 3 h at 45 °C with an enzyme/substrate (E/S) ratio of 10 U/mg protein [197].

**Table 4:** Summary of enzymatic method for protein hydrolysates preparation (adapted from [3]).

Product	Shrimp Waste Sources	Enzyme	Conditions pH–Temperature–Time	DH or DP (%)	Composition of the Free Amino Acids (mg/mL)	Ref.
Flavor protein hydrolysate	*Penaeus chinensis*	Dispase	6.5–57 °C–3 h	57.7 (DH)	Threonine (0.62 mg/mL), valine (1.02 mg/mL), methionine (0.74 mg/mL), lysine (2.58 mg/mL), isoleucine (1.89 mg/mL), leucine (2.76 mg/mL), phenylalanine (1.69 mg/mL), serine (1.74 mg/mL), glutamic acid (2.24 mg/mL), glycine (3.19 mg/mL), histidine (0.39 mg/mL), arginine (4.48 mg/mL), alanine (3.08 mg/mL), proline (0.51 mg/mL), tyrosine (1.85 mg/mL), and aspartic acid (0.89 mg/mL)	[198]
Antioxidant protein hydrolysates	*Metapenaeus dobsoni*	Alcalase	8.2–45.4 °C–3 h	42.4 (DH)	-	[199]
Antioxidant protein hydrolysates	Shrimp byproducts	Alcalase	7.2–NA–NA	NA	serine-valine-alanine-methionine-leucine-phenylalanine-histidine (804.4 Da)	[200]
Protein hydrolysates	*Parapenaeus longirostris*	Alcalase	8–50 °C–30 min	13 (DH)	Asparagine+aspartic acid (98/1000 PFCP), threonine (52/1000 PFCP), serine (56/1000 PFCP), glutamine+glutamic acid (113/1000 PFCP), glycine (128/1000 PFCP), alanine (96/1000 PFCP), cysteine (9/1000 PFCP), valine (56/1000 PFCP), methionine (27/1000 PFCP), isoleucine (48/1000 PFCP), leucine (34/1000 PFCP), tyrosine (34/1000 PFCP), phenylalanine (41/1000 PFCP), hydroxylysine (6/1000 PFCP), histidine (22/1000 PFCP), lysine (75/1000 PFCP), arginine (53/1000 PFCP), and proline (52/1000 PFCP)	[192]
Antioxidant protein hydrolysates	*Farfantepenaeus subtilis*	Alcalase	8–55 °C–30–40 min	3.6 (DH)	-	[117]
Antioxidant protein hydrolysates	*Penaeus monodon* and *Penaeus indicus*	Alcalase	8.5–60 °C–90 min	35 (DH)	-	[118]
Antioxidant protein hydrolysates	Shrimp waste	Digestive alkaline proteinases from *Liza aurata*	8–45 °C–3 h	76 (DP)	-	[197]
Protein hydrolysates	*Litopenaeus vannamei* ^1^	Acid stable protease	3.5–40 °C–6 h	95 (DP)	-	[201]
Protein hydrolysate	*Penaens vannamei*	Endogenous enzyme	9–50 °C–2 h	92.1 (DP) 52.8 (DH)	-	[202]
Protein hydrolysate	*Penaens vannamei*	Endogenous enzyme	8–50 °C–0.4 h	87.5 (DP) 3 (DH)	Depending on the treatment applied, different proportions of histidine, isoleucine, leucine, lysine, phenylalanine, threonine, valine, alanine, arginine, aspartic acid, glutamic acid, glycine, tyrosine, and proline were found.	[203]
Protein hydrolysate	*Litopenaeus vannamei* ^1^	Protease from *Streptomyces griseus*	7–37 °C–3 h	91.1 (DP)	*-*	[97]
Protein hydrolysate	*Litopenaeus vannamei* ^1^	Trypsin or Ficin	7.7 (trypsin) or 7.5 (Ficin)–RT–2 h	92 (DP)	-	[113]
Carotenoprotein	*Litopenaeus vannamei* ^1^	Albacore tuna spleen trypsin	9.5–25 °C–45 min	NA	Alanine (38.58 mg/g sample), arginine (43.80 mg/g sample), aspartic acid/asparagine (71.78 mg/g sample), cysteine (0.21 mg/g sample), glutamic acid/glutamine (81.85 mg/g sample), glycine (54.10 mg/g sample), histidine (28.61 mg/g sample), hydroxylysine (0.66 mg/g sample), isoleucine (32.19 mg/g sample), leucine (52.60 mg/g sample), lysine (39.88 mg/g sample), methionine (16.47 mg/g sample), phenylalanine (35.04 mg/g sample), proline (28.32 mg/g sample), serine (32.97 mg/g sample), threonine (31.88 mg/g sample), tryptophan (8.57 mg/g sample), tyrosine (29.04 mg/g sample), and valine (36.18 mg/g sample)	[114]
Carotenoprotein	*Litopenaeus vannamei* ^1^	Papain	6.82–54.41 °C–48.91 min (shrimp shells) 7.22– 46.23 °C–51.17 min (shrimp heads)	52.45 (shrimp shells; DH) 55.36 (shrimp heads; DH)	-	[115]
Protein hydrolysate	*Litopenaeus vannamei* ^1^	Pepsin	2–40 °C–16 h	92 (DP)	-	[116]

^1^ *Litopenaeus vannamei* and *Penaens vannamei* are synonymous. RT—room temperature; DP—degree of deproteination; DH—degree of hydrolysis.

Fermentation emerges as an eco-friendly and cost-effective alternative process for deproteinization [204]. It operates through proteolytic enzymes generated by microorganisms [205]. Consequently, chitin in solid fraction and liquor with soluble protein hydrolysates, peptides, free amino acids, pigments, phenolics, and antioxidant compounds can be recovered [206,207]. Protein hydrolysates are prepared through lactic acid fermentation of shrimp byproducts. The protein-rich liquid hydrolysates are further processed into a concentrated paste via vacuum evaporation or transformed into a dry powder using a spray-drying method.

Additionally, Antarctic krill juice fermented by *Saccharomyces cerevisiae* was produced with the best flavor and nutrition value. The amino acid content and the scavenging activity of DPPH radicals were up to 1368.3 mg/100 mL and 71.3%, respectively. Moreover, the fermented liquor contained a variety of amino acids, including all eight essential amino acids not manufactured by the human body [206]. In a previous study, the fermentation broth of Antarctic krill with *B. subtilis* OKF04 was analyzed, revealing various bioactive substances, including polypeptides (9.5 g/L), free amino acids (3469.4 mg/L), phenols (1.1 g/L), and polysaccharides (200.8 mg/L) [208].

### 4.2. Application of Protein Hydrolysates

The utilization of synthetic antioxidants has been a longstanding practice; however, concerns regarding their safety frequently arise among consumers. In response, natural compounds exhibiting robust antioxidative properties have garnered increasing attention as viable alternatives. Particularly, researchers have turned their focus to the exploration of byproducts from shrimp waste, recognizing their diverse potential food applications [209]. Protein hydrolysates derived from shrimp waste have diverse potential applications in food technology, serving as flavorings, functional ingredients, or as rich sources of amino acids, making them promising candidates for nutraceuticals in food and pharmaceutical products [210].

Protein hydrolysates from various shrimp species have demonstrated antioxidant activity against lipid peroxidation and reactive oxygen and nitrogen species [117,118,197,199] as well as anti-inflammatory activity [211]. Additionally, Ambigaipalan and Shahidi [212] indicated the ability of shrimp protein hydrolysates to potentially regulate blood pressure by inhibiting the angiotensin I-converting enzyme. The protein hydrolysates obtained by these authors were rich in hydrophobic amino acid residues including alanine, valine, leucine, isoleucine, proline, phenylalanine, and methionine, which can be related to the bioactivity observed. Further enhancing the versatility of shrimp waste-derived protein hydrolysates, Yuan et al. [213] used a Box–Behnken design to optimize shrimp shell waste hydrolysates with α-amylase inhibitory activity. The most antidiabetic hydrolysate was obtained under the following conditions: neutrase concentration of 5.4% (*w*/*w*), liquid–solid ratio of 13 mg/mL, and hydrolysis time of 4.1 h. Under these conditions, the α-amylase inhibitory rate was 43.4%.

Da Silva et al. [214] evaluated the impact of protein hydrolysates obtained from the heads of *L. vannamei* on several parameters related with growth, metabolism, digestibility, and biochemistry in Wistar rats, such as body weight, food and water consumption, urine volume, protein efficiency ratio, food efficiency ratio, net protein ratio, relative net protein ratio, in vivo protein digestibility, apparent digestibility, and biochemical parameters. Compared with the casein group, the diet based on protein hydrolysates proved to be of high biological value, as the rats on this diet presented greater biological responses.

Besides the human diet, shrimp hydrolysates also have the potential to be incorporated into petfood. Guilherme-Fernandes et al. [215] conducted a study with Beagle dogs to compare a commercial diet with diets incorporating 150 g/ kg of squid meal or shrimp hydrolysate. Based on the results obtained for apparent total tract digestibility, metabolizable energy content, fecal characteristics, metabolites, and microbiota, the authors concluded that squid meal and shrimp hydrolysate could constitute novel and promising protein sources for dog food.

## 5. Lipidic Fraction

Shrimp wastes are also a rich source of fatty acids and carotenoids. Different conventional and alternative extraction methodologies have been employed to extract these classes of compounds.

### 5.1. Carotenoids

Valuable pigments, including astaxanthin and its esters, as well as β-carotene, have been identified in various shrimp waste materials [216]. Carotenoids are widely distributed pigments found in various organisms. They are characterized by 40 carbon atoms and conjugated double bonds, making them lipid-soluble tetraterpenoids [217]. They have been effectively extracted from shrimp waste employing conventional and alternative extraction techniques [218]. As for the potential use as a coloring agent, the carotenoids of shrimp waste constitute a widely distributed group of pigments, displaying hues ranging from red to yellow, and blue can also be present, though rarely.

Among the carotenoids, the most present in shrimp waste is astaxanthin [219]. Astaxanthin is naturally produced in freshwater microalgae, particularly *Haematococcus pluvialis* [220]. When these algae face stress due to factors like nutrient deficiency, increased salinity, or excessive sunlight, they synthesize astaxanthin. The presence of carotenoids in crustaceans is mainly attributed to the absorption of pigments from their diet. Animals that consume these algae, including salmon, red trout, red sea bream, flamingos, and crustaceans, such as shrimp, krill, crab, lobster, and crayfish, exhibit the distinctive red-orange pigmentation of astaxanthin [221]. As farmed aquatic animals lack access to natural sources of astaxanthin for achieving the desired reddish-orange coloration, their entire astaxanthin intake needs to be obtained from the feed they are given.

Apart from its role in pigmentation, astaxanthin has been linked to a decreased risk of human diseases, including age-related macular degeneration [222] and ischemic disease [223]. Renowned as a strong antioxidant, astaxanthin surpasses the activity of other carotenoids and vitamin E [224]. Due to its multifaceted bioactivities, including antioxidative, anticancer, immunomodulating, antidiabetic, and anti-inflammatory effects, astaxanthin is widely employed as a coloring agent in aquaculture diets, as well as in the cosmetic and pharmaceutical industries [225].

Shrimp waste is believed to contain heightened levels of high-quality astaxanthin. Owing to its specific binding characteristics, astaxanthin predominantly exists in shrimp waste in conjunction with other compounds, forming a chemical complex with proteins (carotenoproteins) or lipoproteins (carotenolipoproteins) [226].

#### 5.1.1. Conventional Extraction of Astaxanthin

Carotenoids from shrimp wastes are usually extracted with organic solvents. Acetone, ethanol, methanol, dichloromethane, isopropanol, and mixtures of these solvents are commonly employed for total carotenoid extraction or astaxanthin extraction [22,219,227,228,229,230,231,232], as depicted in Table 5.

#### 5.1.2. Green Extraction of Astaxanthin

An alternative approach involves using green technologies, such as extraction with environmentally friendly solvents like vegetable oils [218,227,234,235], supercritical fluid extraction with CO_2_ [234,236,237,238,239], high-pressure techniques [231,240], ultrasound-assisted extraction [228], and fermentation with aerobic bacteria [226].

##### Extraction with Vegetable Oils

Parjikolaei et al. [218] tested two green solvents, sunflower oil and methyl ester of sunflower oil, to extract astaxanthin from *Pandalus borealis* Krøyer processing waste. The influence of temperature (25, 45, 70 °C), solvent/waste ratio (3, 6, 9), waste particle size (0.6 and 2.5 mm), and moisture content (0% and 86.8%) as well as stirrer speed (120, 200, 400 rpm) were studied during the process; the best extraction conditions were 70 °C, solvent/waste ratio of 9, and 400 rpm. Sunflower oil and methyl ester of sunflower oil allowed the extraction of 60% and 80% of astaxanthin, respectively, compared with the conventional extraction process performed with a hexane/isopropanol ratio of 60:40 *v*/*v* (100%). Similar results were found for *Penaeus semisulcatus* De Haan, where authors verified that the conventional organic solvent extraction was more efficient than the extraction with vegetable oils [227].

Messina et al. [234] compared different oil-assisted extraction approaches to obtain the highest yield of astaxanthin from the hydrolysate of *P. longirostris* wastes. The pigment was extracted by different green solvents: crude viscera oil, total fatty acids ethyl esters obtained from crude viscera oil, polyunsaturated fatty acid ethyl esters enriched by short-path distillation, and exhausted fatty acid ethyl esters. CO_2_–supercritical fluid extraction was also employed at 40 °C and 350 bar. Total fatty acids ethyl esters obtained from crude viscera oil proved to be the best extraction solvent, followed by crude viscera oil, and a significant enrichment in astaxanthin by short-path distillation was also verified.

Astaxanthin from *Farfantepenaeus subtilis* Pérez Farfante wastes was extracted with palm olein at temperatures from 50 to 70 °C. A maximum value of 29.81 µg astaxanthin/g of dried waste was obtained [235].

##### Supercritical Fluid Extraction

Messina et al. [234] showed that the supercritical fluid extraction of astaxanthin (40 °C, 350 bar) was not as effective as the vegetable oil assisted extraction in the case of the hydrolysate of *P. longirostris* wastes. The authors suggested that the use of a co-solvent could improve extraction efficiency. Indeed, the extraction of astaxanthin from *P. monodon* waste was improved by the addition of co-solvents, as reported by Radzali et al. [236]. Seven co-solvents were tested (ethanol, water, methanol, 50% (*v*/*v*) ethanol in water, 50% (*v*/*v*) methanol in water, 70% (*v*/*v*) ethanol in water, and 70% (*v*/*v*) methanol in water) at 60 °C and 200 bar, revealing that the ethanol extract produced the highest carotenoid yield (84.02 μg/g dw) with 97.1% recovery (100% achieved by conventional extraction with acetone:methanol (7:3, *v*/*v*)), the highest amount of the extracted astaxanthin complex (58.03 μg/g dw), and the free astaxanthin content (12.25 μg/g dw).

However, in other study, Mezzomo et al. [237] tested the influence of different conditions (with and without co-solvents) to achieve the maximum yield of carotenoids from *Penaeus brasiliensis* and *Penaeus paulensis*. They observed that adding co-solvents (CO_2_ + 2% hexane/isopropanol, CO_2_ + 5% hexane/isopropanol, CO_2_ + 2% sunflower oil, and CO_2_ + 5% sunflower oil) produced extracts with lower carotenoid content (9.12 to 24.2 µg/g) than with pure CO_2_ (1223 µg/g) at 60 °C and 300 bar. The optimum extract was dominated by esterified astaxanthin (768 µg/g extract) followed by β-criptoxanthin (309 µg/g extract) and free astaxanthin (126 µg/g extract), and minor contents of α and β-carotenes.

A Box–Behnken factorial design was applied to the supercritical fluid extraction of astaxanthin from fermented *L. vannamei* wastes. The optimized extract was obtained at 300 bar and 60 °C (astaxanthin concentration of 0.6353 µg/g lyophilized liquor) [238]. Likewise, a central composite design was developed to extract astaxanthin from *P. monodon*, being the optimal extraction condition obtained at T = 56.88 °C and P = 215.68 bar, using ethanol-modified CO_2_ [239].

##### High Pressure

Irna et al. [231] compared the extraction efficiencies of the conventional chemical extraction (with acetone and methanol (7:3, *v*/*v*)) and high-pressure extraction (2100 bar) for a period of 10 min with the same solvent mixture for carotenoid extraction from six species of shrimp, namely *Parapenaeopsis sculptili* Heller, *Metapenaeus lysianassa* De Man, *M. rosenbergii*, *Metapenaeopsis hardwickii*, *Penaeus merguiensis*, and *P. monodon*. The results demonstrated that *P. monodon* was the richest matrix to obtain carotenoids and high-pressure treatment allowed the recovery of higher amounts of total carotenoids (68.26 vs. 46.95 µg/mL) and astaxanthin (59.97 vs. 29.44 µg/mL) than the conventional method.

High-pressure extraction of astaxanthin from *P. vannamei* waste at different pressures (0.1–600 MPa) and holding times (0–20 min), and with different solvents (acetone, dichloromethane, and ethanol) and solvent/solid ratios (10–50 mL/g) was evaluated by Li et al. [240]. Compared with the extraction performed at atmospheric pressure, extractions carried out under pressure were more efficient.

##### Ultrasound-Assisted Extraction

Hu et al. [228] extracted astaxanthin from *Procambarus clarkii* Girard wastes using ethanol as a solvent. UAE was performed at 50 °C, and repeated three times. The obtained extract contained 239.96 μg astaxanthin/g. Dave et al. [229] tested different solvents (acetone, hexane/isopropanol (3:2 (*v*/*v*)), and methanol) to extract astaxanthin from *P. borealis*. Two matrix/solvent ratios were applied, namely 1 g sample:10 mL solvent (for acetone and hexane/isopropanol (3:2 (*v*/*v*)) or 0.240 g sample:10 mL solvent for methanol). The extraction consisted in two steps: 20 s of vortex followed by 5 min UAE at 25 °C. The recovered solid was repeatedly extracted with fresh solvent until the filtrate was clear. Astaxanthin content was highest for hexane/isopropanol (3:2 (*v*/*v*)).

##### Fermentation with Aerobic Bacteria

Cheong et al. [226] optimized the extraction of astaxanthin from a mixture of shrimp shell wastes from different species using *Aeromonas hydrophila*, an aerobic bacteria isolated from shrimp shells. A minimal synthetic media (MSM) consisting of 0.1% (*w*/*v*) K_2_ HPO_4_, 0.05% (*w*/*v*) MgSO_4_·7H_2_O and 9% (*w*/*v*) shrimp shell waste powder was prepared. *Aeromonas hydrophila* were inoculated into the media and incubated at 150 rpm for 48 h at 30 °C. The central composite design determined the optimized culture media conditions to be pH 7.0, monosodium glutamate 3% (*w*/*v*), glucose (1% *w*/*v*), and 30 °C; these conditions allowed them to obtain 1.66 μg/mL of astaxanthin. All the cultures were grown separately in the MSM mixture for 48 h at 150 rpm.

### 5.2. Fatty Acids

Depending on the length of the carbon chains, fatty acids are commonly classified as short-chain (<6 C), medium-chain (6–12 C), and long-chain (>12 C) fatty acids. In addition, fatty acids are divided by the degree of saturation of the carbon chain with hydrogen atoms. Saturated fatty acids (SFAs) have no double bonds, monounsaturated fatty acids (MUFAs) have one double bond, and polyunsaturated fatty acids (PUFAs) contain more than one double bond. Another classification with clinical relevance distinguishes n-3 PUFAs with the end double bond at C3, counting from the methyl end of the hydrocarbon chain, and n-6 PUFAs with the end double bond at C6 [241].

The lipidic fraction of shrimp wastes is mainly composed by fatty acids, being carotenoids in minor amount [219]. SFAs play an important role in the energy metabolism of shrimp. However, excessive SFA intake in humans may increase the risk of cardiovascular disease. MUFAs, such as oleic acid, are also the main energy source in the process of shrimp growth and metabolism. On the other hand, PUFAs are bioactive compounds with a positive impact on the cardiovascular system and their nutritional value is positively correlated with the degree of unsaturated fatty acids [22]. Concerning PUFAs, the WHO/FAO recommends that the ratio n-6/n-3 PUFAS should be 4-6 in the diet for optimal human health [22].

#### Extraction of Fatty Acids

The ethyl acetate extract from *L. vannamei* wastes was chemically characterized by Gómez-Estaca et al. [219]. The most abundant fatty acids were palmitic (C16:0), linoleic (C18:2n6c), oleic (C18:1n9c), DHA (C22:6n3), and EPA (C20:5n3) acids. SFA accounted for 31% of the total amount of fatty acids identified, while MUFA and PUFA represented 25 and 44%, respectively. The same pattern of SFA, MUFA, and PUFA abundance was found for the chloroform/methanol (1:2, *v*/*v*) extract obtained from *Penaeus kerathurus* and *Squilla mantis* wastes [242].

Liu et al. [22] evaluated the presence of fatty acids in the meat, shells, and heads of five species of shrimp (*L. vannamei*, *M. rosenbergii*, *P. monodon*, *Fenneropenaeus chinensis* Osbeck, and *Penaeus japonicus* Spence Bate). Extractions were done with chloroform–methanol solution (2:1, *v*/*v*). The crude fat content in shrimp meat and shrimp shell was low but that of heads was high. SFAs (61.54–73.16 g/100 g) were the main fatty acids, followed by PUFAs (16.26–30.33 g/100 g) and MUFAs (6.05–15.83 g/100 g). The most prevalent SFAs were myristic acid (C14:0, 3.27–10.88 g/100 g), palmitic acid (C16:0, 16.90–32.76 g/100 g), and stearic acid (C18:0, 11.5–25.93 g/100 g). The MUFAs were mainly oleic acid (C18:1) and palmitoleic acid (C16:1), with relative contents ranging from 3.17 to 12.66 g/100 g and 2.08 to 5.38 g/100 g, respectively. PUFAs were mainly EPA (C20:5n-3, 3.08 to 10.29 g/100 g) and DHA (C22:6n-3, 0.33 to 6.44 g/100 g).

The fatty acid composition of *P. longirostris* byproducts was reported by Messina et al. [234]. SFA, MUFA, n-3PUFA, and n-6PUFA were found to account for 19.95, 31.24, 27.92, and 19.50% of the total fatty acids, respectively. EPA corresponded to 9.69% while DHA corresponded to 16.45%.

An extract obtained by Soxhlet extraction from *F. subtilis* wastes showed that the percentages of SFA and PUFA were 43.65% and 56.35%, respectively. The most common SFAs were palmitic (22.05%) and stearic (15.95%) acids, and among MUFAs, the most predominant were oleic acid (11.34%) and palmitoleic acid (8.90%). Concerning PUFAs, EPA and DHA were the most representative, with 12.85% and 10.03%, respectively [235].

### 5.3. Application of Carotenoids and Fatty Acids

#### 5.3.1. Food Coloring

Beyond the bioactive properties of lipid extracts from shrimp, which are going to be discussed in the next subsections, their intense red color, attributed to astaxanthin, makes them excellent candidates for food coloring. With growing consumer concern surrounding synthetic colorants in foods, the adoption of astaxanthin as a natural alternative is increasingly compelling. However, there are some limitations to its use, including its low water solubility, oxidative deterioration during processing and storage, owing to the high degree of unsaturation of PUFAs and astaxanthin, and low bioaccessibility and bioavailability. For this reason, Montero et al. [243] prepared and characterized microcapsules of astaxanthin extracted from *L. vannamei* wastes. Encapsulation by spray-drying resulted in powders with an intense red color and high-water solubility, as well as stability when stored at low temperatures.

#### 5.3.2. Antioxidant Activity

The antioxidant properties of astaxanthin extracted from shrimp wastes are well-documented. Astaxanthin (0.2 nM) from *P. longirostris* extracted using different green solvents (vegetable oils or CO_2_) was tested on a 3T3 cell line exposed to oxidative stress by the chemical inducer H_2_O_2_ (50 µM). From all the tested solvents, only astaxanthin extracted with crude viscera oil was unable to protect the 3T3 cell line from the cytotoxic effects of H_2_O_2_ [234].

Cabanillas-Bojórquez et al. [238] tested the antioxidant capacity of the optimized CO_2_ extract obtained from the lyophilized liquor of fermented *L. vannamei*, obtaining 1.78 ± 0.08 mmol TE/g lyophilized liquor for ABTS, and 5.44 ± 0.47 mmol TE/g lyophilized liquor for ORAC.

Finally, Li et al. [240] showed that the astaxanthin-enriched extract from *P. vannamei* wastes obtained through high pressures displayed better DPPH scavenging activity than the one obtained at atmospheric pressures.

Concerning fatty acids, n-3PUFAs have been shown to reduce mitochondrial dysfunction related to oxidative stress and endothelial cell apoptosis, an effect promoted by an increased activity of endogenous antioxidant enzymes [244].

#### 5.3.3. Anticancer Activity

The anticancer potential of astaxanthin has been extensively documented. In a hamster buccal pouch (HBP) model study, astaxanthin exhibited the inhibition of nuclear factor kappa B (NF-κB) and Wnt signaling by downregulating key regulatory enzymes such as IKKβ and GSK-3β. This effect may be attributed to the blocking of upregulation of signaling kinases Erk/Akt by astaxanthin. Additionally, the anticancer activity of astaxanthin could result from the activation of mitochondria-mediated apoptosis [245].

In the mechanism of action regarding the reversal of mitomycin C (MMC)-induced cytotoxicity in non-small-cell lung cancer (NSCLC) cells (A549 and H1703), astaxanthin was linked to the inhibition of the expression of Rad51 and phospho-AKT (Ser473) protein [246]. Furthermore, astaxanthin was observed to modulate the expression of cell cycle and apoptosis markers, including cyclin D1, Bax, Bcl-2, and p53, inducing cytotoxicity against MCF-7 breast cancer cells. The combined treatment of carotenoids, namely astaxanthin, lutein, and beta-carotene, synergistically inhibited breast cancer progression without affecting epithelial cells [247].

Astaxanthin application inhibited melanoma progression (A375 and A2058) by decreasing the expression of MMP-1, -2, and -9. Additionally, astaxanthin triggered cell cycle arrest in the G1 phase and activated apoptosis-pathway-mediated cell death. In a xenograft model, astaxanthin treatment significantly reduced tumor mass [248]. The nanoemulsion formulation of astaxanthin and tocopherol demonstrated significant cytotoxicity against in vitro cell line models (HeLa, CT26, and T24) [249]. Similarly, the nanoemulsion formulation of astaxanthin and alpha-tocopherol with sodium caseinate blocked cancer cell progression through mitochondrial-mediated apoptosis in in vitro cell lines such as human colorectal (HT-29) and stomach gastric (AGS cells) cancers, activating apoptosis via increased generation of ROS [250].

#### 5.3.4. Neuroprotection Activity

Numerous studies have highlighted the neuroprotective effects of astaxanthin. Astaxanthin pretreatment demonstrated a reduction in L-glutamate-induced cell cytotoxicity, along with a decrease in ROS generation in PC12 cells. The compound also attenuated apoptosis by modulating the Bax/Bcl-2 ratio, caspase-3 expression, and Ca^2+^ influx. Simultaneously, astaxanthin pretreatment inhibited the activation of ROS, accelerated MAPK and NF-κB pathways, and preserved mitochondrial integrity. These findings suggest that astaxanthin exhibits significant neuroprotective activity by modulating multiple pathways in neural cells [251].

The role of astaxanthin in inhibiting neuropathic pain and its mechanisms have been reported. NMDA receptors, particularly NR2A/NR2B proteins, associated with neurogenic pain generation, were affected by astaxanthin. The compound alleviated neuropathic pain by blocking NMDA receptors, particularly NR2B protein. In vitro studies demonstrated that astaxanthin decreased oxidative stress and GFAP (Glial fibrillary acidic protein) expression induced by lipopolysaccharides in C6 glial cells. Importantly, astaxanthin treatment significantly alleviated neuropathic pain behavior in a rat model study [252].

Astaxanthin also showed protective effects against diabetes-associated inflammatory damage to hippocampal neurons. It significantly blocked GFAP expression, caspase-3, IL-6, IL-1β, and COX-2 in a streptozotocin-induced diabetic mice model [253]. GFAP, caspase-3, interleukin-6 (IL-6), interleukin-1β (IL-1β), and cyclooxygenase (COX-2) were used as markers to assess antidepressive effects. Marine-derived astaxanthin and fucoxanthin exhibited neuroprotection against Aβ1-42-mediated toxicity in PC12 neuronal cells. Astaxanthin minimized Aβ1-42-induced cytotoxicity and H_2_O_2_-induced cytotoxicity by downregulating apoptosis in neural cells, leading to better neural outgrowth [254].

The Akt/GSK-3β pathway plays a key role in the occurrence and progression of neurodegenerative disorders by modulating the nuclear factor erythroid 2–related factor 2 (Nrf2) transcription factor. The neuroprotective action of astaxanthin was observed in HT22 hippocampal neural cells by modulating the Akt/GSK-3β pathway, heme oxygenase-1 (HO-1), attenuating caspase activation, and other apoptosis biomarkers, including caspase-3/8/9 activity, PARP (Poly (ADP-ribose) polymerase), and Bcl-2. Astaxanthin treatment also improved apoptosis-inducing factor (AIF), Cyto-c, and Bax proteins [255].

Maternal epilepsy occurrence during pregnancy is mainly associated with brain damage due to oxidative stress. However, treatment with astaxanthin attenuated hippocampal damage in newborn rats by neutralizing oxidative stress, downregulating cAMP response element-binding protein (CREB), and brain-derived neurotrophic factor (BDNF) in hippocampal tissues. Hence, astaxanthin prevents neural damage caused by prenatal maternal epilepsy [256].

#### 5.3.5. Anti-Inflammatory Activity

Astaxanthin treatment demonstrated efficacy in mitigating adverse inflammatory responses in a phthalic anhydride-induced atopic dermatitis animal model and RAW 264.7 macrophage cells. This was achieved by reducing the expression of cyclooxygenase 2 (COX-2), inducible nitric oxide synthase (iNOS), and NF-κB, as well as inhibiting the production of proinflammatory mediators, including TNF-α, IL-6, IL-1β, and IgE [257].

In the context of inflammatory lung injury, astaxanthin exhibited protective effects by inhibiting i-NOS, nitrotyrosine (NT), and NF-κB P65, thus preventing apoptosis-mediated cell death in injured lung tissues [258]. Astaxanthin was reported to lower lipopolysaccharide (LPS)-induced mRNA expression of Il-6 and Il-1β by interfering with the nuclear translocation of nuclear factor NFκB p65. Additionally, astaxanthin successfully decreased LPS-induced oxidative stress while enhancing the nuclear translocation of NRF2 in RAW 264.7 cells. The anti-inflammatory effect of astaxanthin is believed to be mediated through the modulation of the nuclear translocation of NFκB p65 and the inhibition of ROS accumulation in NRF2-dependent and -independent pathways [259].

Moreover, a protective role of astaxanthin on mesenchymal stem cells (MSCs), which are vital for cytokine synthesis including interleukin-6 (IL6), monocyte chemoattractant protein-1 (MCP-1/CCL2), and vascular endothelial growth factor (VEGF), was reported. Astaxanthin treatment helped protect MSCs from palmitate-induced cell death, thereby reducing proinflammatory mediators like IL-6 and subsequent inflammatory pathways [260].

n-3PUFAs, mainly DHA, also display a protective role against inflammation. They can counteract the release of proinflammatory mediators (e.g., thromboxane A2, leukotriene B4, Il-6) in both vascular tissues and in the myocardium, restoring vascular reactivity and myocardial performance [244].

#### 5.3.6. Wound-Healing Activity

The incorporation of astaxanthin-rich carotenoids into formulations has been shown to enhance the wound-healing process by promoting angiogenesis in animal studies. In mice, astaxanthin application demonstrated the ability to overcome cutaneous wound injuries by enhancing the production of tissue-regenerative markers, including collagen type I, alpha 1, and basic fibroblast growth factor (bFGF), and downregulating oxidative stress markers such as nitric oxide synthase (iNOS) at the wound site [261].

A novel approach involved the preparation of an astaxanthin-alpha tocopherol nanoemulsion formulation (ATNE) as an effective formula for wound recovery. ATNE exhibited wound-healing activity in an in vitro monolayer scratch model and correlated with the broad antimicrobial properties of the formulation [249].

A novel formulation called astaxanthin-incorporated collagen film (ACF) exhibited a high percentage of wound-healing activity (71%) in both full-thickness excision and linear incision in rats. ACF treatment showed positive signs of wound healing, including high collagen content, granulation, fibroblasts, thickened scar development, improved neovascularization, and rapid epithelialization within a short duration [262]. Fang et al. [263] reported that astaxanthin treatment successfully prevented the early progression of burn wounds in a classic “comb” burn rat model study. Astaxanthin treatment reduced oxidative stress during the early stages of burns by activating the NADPH-dependent oxidase system, enhancing the activity of SOD and GPx, and lowering the expression of inflammatory mediators such as MPO, IL-1β, IL-6, and TNF-α in the zone of stasis. Astaxanthin downregulated the activation of the PI3K/Akt/Bad/caspase signaling cascade, thereby accelerating mitochondria-related apoptotic cell death in the zone of stasis. The conclusion was that astaxanthin prevented early burn-wound progression by regulating antioxidant, inflammatory, and apoptosis pathways [263].

#### 5.3.7. Hepatoprotective Activity

Astaxanthin has demonstrated potential hepatoprotective activities. Astaxanthin treatment reversed acetaminophen-induced liver injury and associated pathological changes in male C57BL/6 mice by enhancing GSH and SOD levels. Moreover, astaxanthin interfered with apoptosis changes by blocking the TNF-α-mediated JNK signal pathway and modulating the phosphorylation of ERK and P38, thereby preventing liver damage [264]. An attempt was made to assess the ability of astaxanthin to prevent fibrogenic effects of TGFβ1 in hepatic stellate cells (HSCs) using LX-2 cells, an immortalized human HSC cell line. Astaxanthin treatment significantly lowered the expression of TGFβ1-induced α-smooth muscle actin (α-SMA) and Col1A1, as well as α-SMA protein levels. Astaxanthin inhibited TGFβ1-induced Smad3 phosphorylation and subsequent expression of Smad3, Smad7, TGFβ receptor I (TβRI), and TβRII proteins. Evidence suggests that astaxanthin exhibited anti-fibrogenic effects by inhibiting TGFβ1 signaling and concomitantly inhibiting the activation of the Smad3 pathway in HSCs [265]. The regulatory role of astaxanthin on hepatic lipid accumulation in high-fat-fed C57BL/6J mice was investigated. Astaxanthin ameliorated fatty acid metabolism by upregulating PPAR α. Astaxanthin modulated SREBP-1 phosphorylation and lowered subsequent lipogenesis in liver tissue. Astaxanthin reduced hepatic steatosis through various mechanisms, including regulating PPARα and PPARγ, blocking Akt activity, and stimulating hepatic autophagy [266].

#### 5.3.8. Antidiabetic Activity and Cardiovascular Protection

Thrombotic risk is considered a major problem associated with type 2 diabetes. Astaxanthin administration has been shown to decrease diabetes-associated coagulation disorders [267]. Moreover, astaxanthin administration ameliorated metabolic events and lowered blood pressure associated with type 2 diabetes [268].

Saini et al. [269] compared the fatty acid profiles of three shrimp species: *P. monodon*, *P. vannamei*, and *Pleoticus muelleri* Bate. The obtained results were used to calculate several nutritional parameters of lipids, including ratios of PUFAs/SFAs, PUFAs/MUFAs, hypocholesterolemic (h)/hypercholesterolemic (H) fatty acids, the atherogenic index (AI), and the thrombogenic index (TI). The PUFAs/SFAs ratios ranged from 0.89 (*P. monodon* waste) to 1.70 (*P. muelleri* waste), being, therefore, greater than 0.45, which is the recommended lower limit for human consumption to minimize the risk of cardiovascular diseases and other chronic diseases. TI varied from 0.18 (in *P. muelleri*) and 0.46 (in *P. monodon*), while AI was in the range of 0.25 and 0.42. These low values demonstrate the cardiovascular protection displayed by shrimp waste extracts, since the AI value expresses a pro-atherogenic activity and the prevention of the anti-atherogenic effect and TI expresses the tendency to form clots in the blood vessels. Moreover, *P. muelleri* presented the highest occurrence of EPA and DHA, the highest total amount of n3-PUFAs (34.3% in waste and 38.2% in edible flesh), and the highest ratio of n3/n6 PUFAs (4.03 in waste and 5.65 in edible flesh).

## 6. Materials and Methods

A search of papers published between 2013 and 2024 was performed based on several keywords and their combinations: “Shrimp wastes”, “Green Extraction”, “Enzymatic Extraction”, “Green Recovery”, “Chitin”,” Chitosan”, “protein hydrolysates”, “Astaxanthin”, “PUFAs”, “Seafood Industry”, “Shrimp wastes bioactivities”, “Microwave-assisted extraction”, “ultrasound-assisted extraction”, “subcritical water extraction”, “supercritical fluid extraction”, and “biological extraction”. The selection of papers was performed according to the following criteria: (1) only papers written in English were considered; (2) papers dealing with the valorization of shrimp wastes were included; (3) only papers describing the extraction methodology while presenting the full characterization of the extracts obtained and/or bioactivities were included.

## 7. Conclusions

**Figure 4:**
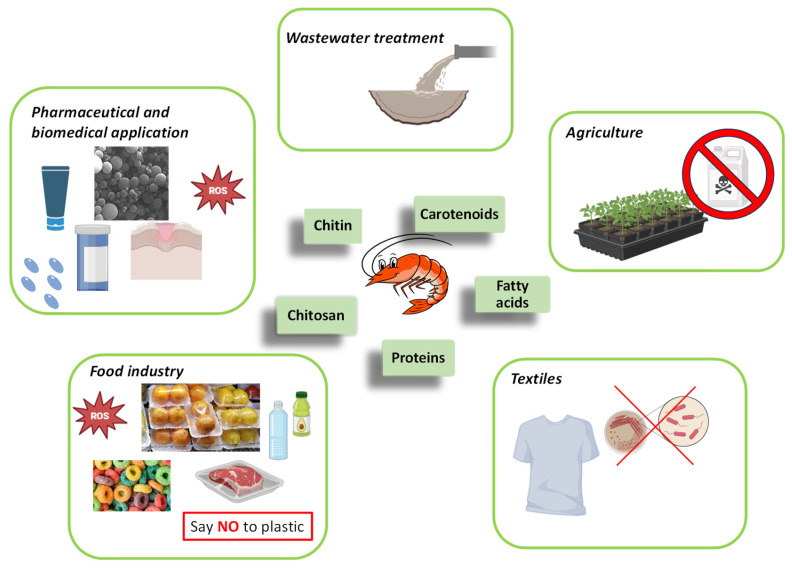
Applications of shrimp waste, reviewed and discussed in this paper. Shrimp wastes are rich in chitin, proteins, carotenoids, and fatty acids, which have several possible applications in different fields, such as the food and pharmaceutical industries, wastewater treatment, agriculture, and textiles.

The valorization of shrimp waste presents a promising avenue for sustainable resource utilization and the extraction of valuable bioactive compounds. Figure 4 highlights the potential applications of shrimp waste in various industries. Through this review, it becomes evident that shrimp waste is not merely a byproduct of the seafood industry but a potential reservoir of polysaccharides, proteins, and lipids and carotenoids, with diverse applications across various sectors. Polysaccharides, primarily chitin and chitosan, extracted from shrimp waste exhibit a wide range of beneficial properties, holding promise for applications in pharmaceutics, biomedicine, agriculture, and environmental remediation, among others. Moreover, the adoption of green extraction techniques enhances the sustainability of the process, minimizing environmental impact. Protein hydrolysates derived from shrimp waste offer additional opportunities as bioactive compounds and functional ingredients. Their extraction and utilization in various applications, such as food and pharmaceuticals, highlight the potential for value-added products from shrimp waste. Carotenoids, particularly astaxanthin, extracted from shrimp waste, demonstrate not only possible applications as food colorants, but also remarkable antioxidant, neuroprotective, and anti-inflammatory activities, among others, with potential applications in health and wellness products. Finally, PUFAs are well recognized due to their cardiovascular protective effects, particularly omega-3 fatty acids.

In conclusion, the valorization of shrimp waste not only addresses environmental concerns associated with waste disposal but also unlocks significant economic and societal benefits. Continued research and development in extraction techniques, coupled with innovative applications, will further enhance the value proposition of shrimp waste utilization. Ultimately, leveraging shrimp waste as a resource contributes to the broader goal of achieving sustainability and circular economy principles in the seafood industry.

## Figures and Tables

**Table 1:** Application fields and potential uses of chitin and its derivatives.

Field	Example of Application	Ref.
Pharmaceutical	- Excipients and drug carriers - Drug/gene delivery - Cancer diagnosis and treatment	[30,31,32,33,34]
Biomedical and biotechnology	- Scaffolds fabrication for tissue regeneration - Three-dimensional cell culture systems - Barrier of chitin-based dressings for microbial infections - Surgical stitches development - Hemostatic dressing - Enzyme immobilization - Biosensors manufacturing	[35,36,37,38,39,40,41,42,43]
Cosmetic and cosmeceutical	- Vehicle for active ingredients in skin care	[44,45]
Food and nutrition	- Emulsifying, fining, thickening, and stabilizing agents, antioxidants, and low-calorie food mimetics - Composite films/coatings - Food preservation	[46,47]
Textile	- Antimicrobial and non-allergenic fibers - Agents for dyes and chemicals removal from textile wastewaters	[48,49]
Industrial	- Adsorbents for removing heavy metals from water - Biomaterial for industrial packaging	[50,51]
Agriculture	- Plant root growth enhancer - Plant protection (antimicrobial, antifungal, and antiviral activities)	[52]
Papermaking industry	- Retention and drainage agents, paper strength agents - Coating agents	[19]

**Table 2:** Comparison between chemical and biological methods for demineralization and deproteinization steps [26,110].

Extraction Methods	Treatment	Advantages	Disadvantages	Quality of Chitin
Traditional chemical extraction	**Acidic treatment**: Demineralization with decalcifying agents (HCl, HNO_3_, H_2_SO_4_, CH_3_COOH, and HCOOH). **Alkali treatment**: Deproteinization with NaOH.	- Used at the industrial scale for chitin preparation. - Short processing time.	Environmentally unfriendly; high cost because of the effluent treatment generated after acid and alkaline reagents. Removed proteins and minerals cannot be used as human and animal food supplements after being in contact with the acidic and alkali treatment.	Despite the complete removal of organic salts, there can be deacetylation and depolymerization reactions.
Biological extraction	**Lactic acid treatment**: Demineralization with lactic-acid-producing bacteria. **Proteases treatment**: Deproteinization with proteases-producing bacteria.	- Environmentally safe and low-cost because of the absence of effluent. - Solubilized proteins and minerals may be used as human and animal nutrients.	Limited to laboratory scale studies only; excessive time of process; need for more complex experimental devices.	Homogeneous and high-quality final products.

**Table 3:** Mechanisms of the anticancer activity of chitosan and derivatives.

Mechanism of Anticancer Activity of Chitosan and Derivatives	Description	Ref.
Permeation enhancer	Amino group in chitosan leads to protonation in acidic–neutral medium. The positive charge developed makes chitosan water soluble and bioadhesive to bind with and enhance permeation through negatively charged epithelial surfaces. Therefore, chitosan can enhance the passage of polar drugs through epithelial surfaces.	[145,146,147]
Antiangiogenic effect	Chitosan oligosaccharides have an inhibitory effect on angiogenic activities of HUVECs. Chitosan oligosaccharide inhibited cell proliferation activity, cell migration, and vascular endothelial cell growth factor (VEGF) expression of human umbilical vein endothelial cells. VEGF is a potent angiogenic factor.	[145,148,149]
Immune enhancer	Chitosan demonstrates immune enhancement through two distinct mechanisms. Firstly, acting as an immune adjuvant, it triggers immune system activation, leading to the generation of proinflammatory factors. Secondly, it serves as a carrier for immunotherapeutic drugs, facilitating targeted delivery and enhancing bioavailability while mitigating systemic toxicity.	[145,150]
Apoptotic effect	Low-molecular-weight chitosan can induce G1/S cell cycle arrest and caspase activation in cell lines.	[145,151,152]

**Table 5:** Conventional extraction of carotenoids from shrimp wastes.

Shrimp Waste Sources	Type of Extraction	Extraction Conditions	Observations	Ref.
*Pandalus borealis*	Extraction with hexane/isopropanol, (60:40 *v*/*v*)	5 g sample:25 mL solvent; repeated 4 times	41.1 mg astaxanthin/Kg wet waste material	[218]
*Litopenaeus vannamei*	Stirring with ethyl acetate	10 g sample: 50 mL solvent; stirred for 30 min at room temperature in darkness	7 mg astaxanthin/g lipid extract	[219]
*Penaeus semisulcatus*	Stirring with different solvents (acetone, hexane, acetone/hexane (50:50), isopropyl alcohol, isopropyl alcohol/hexane (50:50))	10 g sample: 20 mL solvent; stirring (2 min) at room temperature	Best extraction yield with acetone (61.3 µg carotenoids/g waste)	[227]
*Litopenaeus vannamei*, *Macrobrachium rosenbergii*, *Penaeus monodon*, *Fenneropenaeus chinensis*, *Penaeus japonicus*	Agitation with an oscillator using dichloromethane/methanol (1:3, *v*/*v*) as a solvent	200 mg: 5 mL solvent, agitation for 3 h	From 2.91 μg astaxanthin/g (*Penaeus monodon*) to 19.20 μg astaxanthin/g (*Litopenaeus vannamei*)	[22]
*Litopenaeus vannamei*	Blending with ethanol	500 g sample: 1000 mL solvent.	28.9 mg astaxanthin/g shrimp shells	[230]
*Parapenaeopsis sculptili*, *Metapenaeus lysianassa*, *Macrobrachium rosenbergii*, *Metapenaeopsis hardwickii*, *Penaeus merguiensis*, and *Penaeus monodon*	Mixing with acetone/methanol (7:3, *v*/*v*)	1 g sample: 5 mL solvent at room temperature	*P. monodon* contained the highest total carotenoid (46.95 µg/mL) and astaxanthin (29.44 µg/g dw) content	[231]
*Litopenaeus vannamei*	Mixing with ethanol	Raw materials were mixed with ethanol (raw material/ethanol, 1:2 *w*/*v*); the extraction was repeated in triplicate	The yields of astaxanthin from fresh shrimp heads, cooked shrimp heads, fresh shrimp shells, and cooked shrimp shells were 3.64, 2.38, 14.65, and 11.76 mg/g crude extract	[232]
*Palaemon serratus* and *Palaemon varians*	Stirring macerations with different solvents (ethanol, 50% ethanol and 70% acetone), different solid/solvent ratios (5 g:50 mL and 5 g:100 mL), and different temperatures (room temperature and 40 °C) were tested	The optimal condition was 5 g of shrimp shell and 50 mL of absolute ethanol solution under agitation for 2 h at room temperature	The mean values ranged between 1.1 and 26.1 μg/g dw	[233]

## Data Availability

The data are contained within the review.
